# Cost of implementing CAR-T activity and managing CAR-T patients: an exploratory study

**DOI:** 10.1186/s12913-023-10443-5

**Published:** 2024-01-22

**Authors:** Maria Caterina Cavallo, Marianna Cavazza, Francesca Bonifazi, Beatrice Casadei, Ilaria Cutini, Barbara Tonietti, Riccardo Saccardi, PierLuigi Zinzani, Claudio Jommi

**Affiliations:** 1 Cergas, Centre for Research on Health and Social Care Management, SDA Bocconi School of Management, Bocconi University, Via Sarfatti, 10, Milano, 20136 Italy; 2 IRCCS Azienda Ospedaliero-Universitaria di Bologna, Via Albertoni 15, Bologna, 40138 Italy; 3 Azienda Ospedaliero-Universitaria Careggi, Largo Brambilla 3, Firenze, 50134 Italy; 4 Department of Pharmaceutical Sciences, Università del Piemonte Orientale, Largo Donegani, 2, Novara, 28100 Italy

**Keywords:** CAR-T, Organisational impact, Cost impact, Activity-based costing, Italy

## Abstract

**Background:**

Chimeric antigen receptor T cells (CAR-T) represent an innovation but raise issues for healthcare payers because of the uncertainty on impact at market launch, high cost and important organisational impact. The literature has focused on their assessment, appraisal and market access solutions. No evidence on the costs sustained to implement CAR-T is available and a few studies reported the cost of the CAR-T clinical pathway, including the activities that are remunerated through inpatient or outpatient fee-for-service/episode. This paper aims at filling the information gap, assessing the cost of implementing CAR-T activity and the full cost of managing the CAR-T clinical pathway.

**Methods:**

Cost analysis relied on the Activity Based Costing approach, which was applied to two Italian healthcare organisations, both CAR-T Centres authorized by the regional governments with a minimum of 20 patients treated with the first two CAR-T therapies launched on the market.

**Results:**

The cost of implementing CAR-T was estimated at €1.31 million (calculated for one of the organizations with complete data). Most of these costs (77%) were generated by quality assurance activity. The mean cost per patient entering the CAR-T pathway (59 and 27) and surviving at follow-up (21 and 5) ranges from €48K to €57K and from €96K to €106K, respectively. Fees for hospitalization and infusion of gene therapy accounts for more than 70% of these costs. The actual hospitalisation cost varies greatly across patients and is in general lower than the fee-for-episode paid by the region to the hospital.

**Conclusions:**

Despite its limitations (exploratory nature; the time spent by staff on activities which are not remunerated through fees was estimated through interviews with the CAR-T coordinators; cost items are not fully comparable), this research highlighted the relevant organisational and economic impact of CAR-T and provided important insights for policy makers and healthcare managers: the necessity to invest resources in CAR-T implementation; the need for assessing activities which are not remunerated through fees for service / episode; the opportunity to shift from fee-for-episode / service to bundled payments for CAR-T clinical pathway.

## Background

Chimeric antigen receptor T cells (CAR-T) are lymphocytes where T cell receptors have been modified and equipped to recognize and destroy malignant tumoral cells via a specific receptor [1]. They represent an innovation in the treatment of patients suffering from haematological malignancies with very poor prognosis, having previously failed standard therapies [2]. Lympho-monocytes from peripheral blood of candidate patients are harvested by a leukapheresis and then sent to a Good Manufacturing Practice (GMP) approved cell factory for transduction and expansion in a GMP-approved facility. After manufacturing, the cellular product is shipped back to the patient hospital for infusion after a lymphodepleting therapy.

The first applications of CAR-T occurred in patients with resistant/relapse acute lymphoblastic leukaemia (ALL) [3] and several subgroups of B cell lymphomas [4], using the CD19 antigen as the tumoral target for immunological destruction. Approval was granted to axicabtagene ciloleucel and tisagenlecleucel, based on results from the first two pivotal studies, ZUMA-1 [5, 6] and JULIET [7]. Three anti CD19 CAR-T cell products are currently available in Italy, as third or subsequent line treatment, for refractory/relapsed ALL (tisagenlecleucel), third line treatment for diffuse large B cell lymphomas (either de novo or transformed), high grade B cell lymphomas, transformed follicular lymphomas (axicabtagene ciloleucel, and tisagenlecleucel), primary mediastinal B cell lymphomas (axicabtagene ciloleucel) and for mantle cell lymphomas (brexucabtagene autoleucel) [8]. The field of CAR-T research is growing rapidly from a variety of biotech, private companies and academic institutions. In April 2022 more than 2700 studies with advanced cellular therapy agents in the global immune-oncology pipeline were ongoing, mainly in haematological malignancies [9].

CAR-T, and cell and gene therapies in general, pose issues for healthcare payers in all jurisdictions where they have been evaluated or are under evaluation. Literature is abundant on distinctive characteristics of cell and gene therapies from a Health Technology Assessment (HTA) viewpoint [10–15], and on how these characteristics might shape price and reimbursement negotiations [16–18]. Critical aspects of gene and cell therapies for HTA include [19]:

- marketing authorisation based on accelerated approval pathways, which allows for trials using surrogate rather than clinical endpoints where, since these medicines are mostly for rare conditions, higher standards of validation are difficult to achieve;
- high preponderance of single arm, uncontrolled clinical studies, either because the patient population is small, or because of practical difficulties or ethical concerns in randomization due to the potential improvement offered by the new therapy;
- small sample sizes that greatly increase the uncertainty around clinical effect size, and also mean that any heterogeneity in the patient population is hard to analyse;
- clinical studies of short duration at the time that HTA is carried out, with difficult extrapolation of the long-term impact.

CAR-T, like virtually all gene and cell therapies, raise financial sustainability issues since they are often very costly. The uncertainty of the evidence at market launch and their potentially considerable financial impact have often made cell and gene therapies a prime candidate for Managed Entry Agreements, also known as performance-linked reimbursement, coverage with evidence development and staged payments [16, 20, 21].

CAR-T are also complex to manage for healthcare organisations. These technologies require the identification of healthcare centres with appropriate organisational structures and competencies, which must be provided with the resources necessary to implement and manage CAR-T activity.

In Italy, CAR-T centres should be equipped with JACIE (Joint Accreditation Committee - ISCT & EBMT) accreditation for allogenic transplant, an intensive care unit and the presence of a multidisciplinary team [8]. Regional governments authorize the healthcare centres, that contract afterwards with the companies holding the marketing authorisations for CAR-T, and provide for remunerating inpatient and outpatient care through fee-for-episode and fee-for-service, respectively [22]. The number of accredited centres is very different across regions, reflecting not only the expected dimension of the target population, but also political choices [23]. In 2022 there were 111 accredited centres, but only 65 (59%) were active (i.e. with almost one patient treated). In the North-Centre of Italy 71% were active, whereas in Southern Regions only in 34% of accredited centres almost one patient was treated. The number of active centres per one million inhabitants ranges from 1.4 in Northern Regions to 0.7 in the Southern ones and more than 50% of CAR-T was administered in 4 out of the 21 Regions, with an average 6 patients treated per centre (ranging from 3 to 20) [23]. According to a recent analysis, the patient access to CAR-T in Italy is lower than in the other largest EU countries [24].

Dedicated funds for implementing CAR-T and managing CAR-T patients are quite rare in Europe. Among the five largest European countries only Germany, through the NUB system (Neue Untersuchungs und Behandlungsmethoden - New examination and treatment method), provides hospitals with dedicated funds for health technologies with an important organisational impact [8].

The organisational and economic impact of CAR-T and the complexity of the associated clinical pathway have received scant attention not only from payers but also in the literature, although they are cited as the reason for delays in patient access, at least as important as long and complex price and reimbursement negotiation [8].

Recently, some papers partially addressed the cost of managing CAR-T beyond the treatment acquisition costs. These papers were elicited from a rapid non-systematic review of the literature on Pubmed and Google Scholar, using the following key-words: CAR-T or CAR T, cost(s), and economic or organisational impact and clinical pathway.

A first set of U.S. publications designed scenarios of clinical pathways on the grounds of clinical studies, the literature evidence, clinicians’ opinion and monetized them, using fee-for-service / episode [25–27]. One paper estimated costs for 551 patients from 6 months before CAR-T-cell administration to 11 months of follow-up on average on the grounds of Medicare administrative data, including inpatient, outpatient, and emergency department services [28]. As far as Europe is concerned, one German paper raised the awareness on the complexity and interdisciplinary nature of the clinical pathway for CAR-T, advocating a proper procedures’ coding and compensation, but not did estimate the cost of this pathway [29]. Another paper focused on administrative data collected during the hospital stays from the French Medical Information Systems Program (PMSI) [30]. The cost of clinical pathways was presented in two papers. The first study collected data for 20 patients in in one Portuguese centre, including inpatient and outpatient services in four stages (from referral date to CAR-T-cell therapy until the day before infusion; from infusion day until day 30 after infusion; between 31 and 60 days after infusion, and between 61 and 150 days after infusion) [31]. Costs of other activities not individually provided to patients were not included. The second study was carried out in Italy [32]. The authors, through an Activity Based Costing (ABC) approach, estimated the cost of the clinical pathway of 47 CAR-T patients over a 36 months’ time horizon, including all activities remunerated trough fee-for-service and side effect management.

None of the previous studies included activities not remunerated through fees and the cost of implementing the CAR-T treatments. Moreover, the Italian paper did not compare the fees for inpatient services with the estimated costs.

This paper aims at filling this information gap, by:

- analysing the organizational model adopted to implement CAR-T in clinical practice and manage the relevant patients, from the identification of eligible patients to follow-up (clinical pathway). The implementation of CAR-T includes the following activities: internal staff and referral centre staff training, quality assurance activities and drafting of Standard Operating Procedures (SOP), protocols and check lists;
- estimating the costs of implementing CAR-T in clinical practice and managing the clinical pathway, excluding the acquisition costs of commercial CAR-T.

Our secondary objectives was to estimate the proportion of costs of the clinical pathway covered by fees provided by the Italian regional governments, and to compare for each inpatient episode, actual costs with fees.

## Methods

Cost analysis relied on an ABC approach [33]. ABC requires the identification of all activities involved in the implementation of CAR-T and the management of the relevant clinical pathway, the organisational units and individuals who carry out these activities, the relationships between organisational units, activities and final output, and the estimation of full cost, i.e. resources and unit costs.

The ABC approach was used for three reasons. First, CAR-T cost estimates are complex since diverse organisational units and professionals are involved. Furthermore, the patient journey is managed in different settings (outpatient services, day hospital and inpatient hospitalization). Finally, ABC provides the opportunity to identify the cost drivers of each activity, which may support actions aimed at making the implementation in other healthcare centres and the clinical pathway more efficient.

ABC was conducted in two large healthcare organisations, a public clinical research and teaching hospital - Azienda Ospedaliero-Universitaria di Bologna (IRCCS AOU BO Orsola, hereafter) and a public teaching hospital - Azienda Ospedaliero-Universitaria Careggi di Firenze (AOU Careggi hereafter). These two centres were selected based on the availability to collect data [34] among CAR-T Centres designated by regional governments with a minimum 20 patients treated with idecabtagene vicleucel and ciltacabtagene autoleucel. A third private clinical research hospital agreed to join the project, but was not able to collect data.

The adopted perspective is that of the healthcare organisations involved. Activities provided by other healthcare organisations were excluded.

Clinical pathway costs refer to period of time between the first patient reported as potentially eligible for CAR-T by the referring centre (September 2020 at the IRCCS AOU BO Orsola and March 2020 at the AOU Careggi) to September 2021.

ABC analysis relied on both primary and secondary data collection. Secondary data include all data that are routinely collected by healthcare organisations, i.e. activities which are awarded a fee-for-service or fee-for-episode, unit cost per hospitalisation day in inpatient wards and intensive care units, which were used to estimate the cost of the hospitalisation episode, and the gross salary of healthcare professionals.

**Fig. 1:**
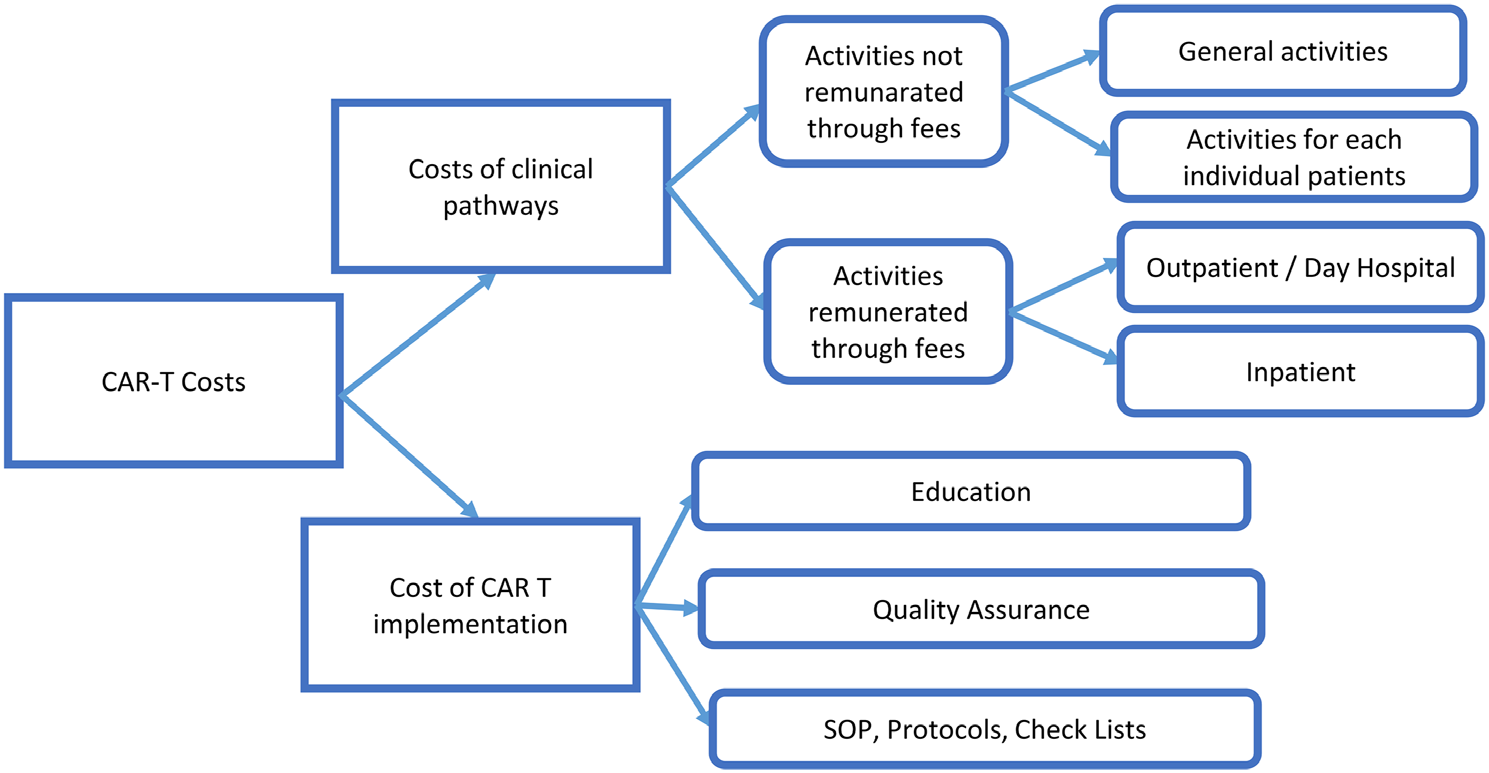
Data collection clusters for CAR-T activity. Source: Primary data collection (interviews with the CAR-T coordinators)

Primary data were collected through an open interview administered to the CAR-T Team coordinators at each hospital. They include (Fig. 1):

- CAR-T implementation activities;
- services provided to individual patients but not remunerated through fees (for example, coordination meetings with the referral centres);
- other activities (for example, coordination of the CAR-T Team or updating procedures, protocols and check lists).

The administration of an interview to the coordinators of the CAR-T Team was preferred to other data collection methods. Time-motion technique, considered the most effective in the literature [35], was not manageable because of the high workload of the healthcare professionals involved, and possibly less robust, since time-management was highly affected by the Covid-19 pandemic. For the same reasons we decided not to rely on questionnaires administered to each individual person involved in CAR-T activities. Coordinators were more likely to provide complete, consistent and unbiased responses.

Overhead costs were proportionally allocated to direct costs of CAR-T activities. Overhead costs estimates were provided by the cost accounting unit of the two healthcare organisations and range from 14.5% at the IRCCS AOU BO Orsola to 26% at the AOU Careggi.

Both primary and secondary data were entered into an Excel database. The database was structured to collect new data on already recruited patients (e.g. follow-up data) and on newly enrolled patients.

All data were validated by the coordinators of the two healthcare organisations. The hospitalization cost and its comparison with the fee-for-episode was carried only at IRCCS AOU BO Orsola, where data on the cost per hospitalisation stay (inpatient wards and intensive care unit) and diagnostic services provided to each hospitalised patient was available.

## Results

As mentioned before, the implementation activities and clinical pathway activities which are not remunerated through fees were elicited from the interviews with the CAR-T activity coordinators.

Table 1 illustrates the activities not remunerated by fees and Table 2 reports the organizational units and professionals involved in the CAR-T team.

**Table 1:** CAR-T clinical pathway: activities not remunerated by fees

PHASE I: PATIENT ELIGIBILITY
Waiting list management
Patient eligibility evaluation and referral center communication
Evaluation protocols
PHASE II/III: PATIENT CONSENT + DRUG CHOICE
Consent form management
Drug choice
Communication to the relevant pharmaceutical companies
Contact with the apheresis transfusion service
First contact with the intensive care unit
PHASE IV: APHERESIS PLAN + DRUG BOOKING
Apheresis planning
Entering drug request in the company’s platform
Apheresis protocol
PHASE V: APHERESIS
Eligibility check
PHASE VI: BAG SENDING + LYMPHOCYTE ENGINEERING PROCEDURE + BAG RETURN
Bag shipping
Contact with the company for engineering project monitoring
Bag reception, preparation and planning
Transplant program checklist
Pharmacy, surgery and cryopreservation checklist
PHASE VII: ADMISSION PLANNING
Pre-hospitalization patient assessment
Pre-admission checklist
Infusion preparation and administrative activities (including compiling drug registry)
PHASE VIII: ADMISSION / RECEPTION OF THE BAG
First contact with the intensive care unit
Lymphodepleting chemotherapy check
Return of modified leukocytes
PHASE IX: HOSPITALIZATION / INFUSION + MONITORING
Protocols / SOPs
PHASE X: HOSPITAL / TOXICITY MANAGEMENT
Toxicity management protocols
Activation of beds in other units
PHASE XI: DISCHARGE
Preparation of the discharge documents
PHASE XII: FOLLOW UP
Protocols for managing toxicities
Compiling follow-up drug registry form

Source: primary data collection (interviews with the CAR-T coordinators)

**Table 2:** Organisation Units and Professionals involved in the CAR-T team (2021)

IRCCS AOU BO Orsola*	**Healthcare Professionals**	**#**
L.A. Seràgnoli Institute of Hematology and Medical Oncology	Hematologist	8
	Nurse	5
	Biologist	4
	Administrative Staff	1
Immunohaematology and Transfusion Unit	Transfusionist	2
Clinical Pharmacy	Pharmacist	3
Anesthesiology and intensive care unit	Anesthesiologists	2
Urgency-Emergency (neurological support)	Neurologist	2
Total		**27**
**AOU Careggi**	**Healthcare Professionals**	**#**
Department of Cellular Therapies / Transfusion	Hematologist/Transplantologist	5
	Transfusionist	2
	Nurse	1
Bone marrow transplant laboratory	Biologist	7
Stroke Unit	Neurologist	1
Department of oncological anesthesia and intensive care	Intensivist	2
Department of Cardiology	Cardiologist	2
Department of Infectious and tropical diseases	Infectious disease specialist	2
Department of Nuclear Medicine	Nuclear radiologist	1
Department of Radiodiagnostics	Radiologist	2
Department of Radiotherapy	Radiotherapist	1
Department of Pharmacy	Pharmacist	1
Total		**27**

Source: Primary data collection (interviews with the CAR-T coordinators)

* In 2022 two infectious disease specialists, three nuclear radiologists, two neuroradiologists, one anatomo-pathologist and one psychologist joined the interdisciplinary team

At the IRCCS AOU BO Orsola the CAR-T team is composed of 27 healthcare professionals including disease-specific hematologists, transplant-dedicated hematologists and non-hematological consultants to cover all complication-specific fields. The activation of the CAR-T cell team begins from the identification of a candidate patient on behalf of the disease-specific group. The patient is put into the electronic waiting list. The transplant team organizes the apheresis in a standardized, JACIE- validated way while the disease-specific group evaluates the need and the type of a possible bridging therapy. The patient is admitted to the transplant unit for the lymphodepletion, infusion of CAR-T cells and management of acute adverse events under the responsibility of the transplant unit. After discharge the patient returns to the disease-specific group for follow-up and long term complications management. During the first phases of the process pharmacists are informed and involved in several steps, including the request for CAR-T cells, cellular product accountability and check of the correct status of cells at delivery (because the cells cannot stay in the pharmacy since the storage is in the processing lab of the transplant team with an authorized cryobank), and for the administration of specific drugs and pharmacovigilance requirements. Similarly, intensivists and neurologists are informed and involved from leukapheresis until admittance into the transplant ward, and after infusion they intervene regularly regarding patient status. After discharge, the competent authority requires that patients stay within two hours’ distance from the infusion centre; for those patients who reside elsewhere, a non-profit organisation hosts them for free in an appropriate facility. Follow-up visits and exams at pre-specified time points are carried out at the IRCCS AOU BO Orsola in accordance with the referral centres for one year. The major effort of the CAR-T cell team is the communication among all actors and the traceability of the interface, which is under strict control by checklist and process indicators.

The CAR-T team at the AOU Careggi is composed of 27 healthcare professionals. The eligibility of a candidate is discussed at a weekly meeting by the CAR-T team, comprising a disease specific hematologist consultant, the CAR-T coordinator (a hematologist expert on immune effector cells therapy), a cardiologist, an infectious disease specialist, a radiotherapist, and radiologists. After assessing eligibility, the CAR-T coordinator books the leukapheresis appointment according to the slot given by the pharmaceutical company, the apheresis specialist, the pharmacists and the cell processing unit. After the leukapheresis, the CAR-T team discusses the need to administer a bridging therapy. Before proceeding to lymphodepleting, three aspects are considered: the availability of CAR-T cells in the processing unit, the availability of anti-cytokines therapy for the treatment of Cytokine Release Syndrome (CRS) in the clinical unit and the confirmation of the patient’s eligibility. Each patient is evaluated by both a neurologist and an intensive care consultant before the administration of lymphodepleting therapy either in the outpatient clinic or after the hospital admission. Every day after the infusion, the CAR-T coordinator sends an e-mail to both the neurologist and an intensive care consultant to notify them of the patient’s clinical status. Both consultants are paged in case of moderate grade of either CRS or Immune effector Cell-Associated Neurotoxicity syndrome occurs. After discharge, the patient is monitored in the outpatient clinic for intermediate and long-term side effects: infections, hematological and non-hematological toxicities. Each time point disease evaluation is discussed by the CAR-T team during the weekly meeting as well as the need for subsequent therapy in case of failure. After one year of follow up under the responsibility of the CAR-T center, the patient returns to the referring hematologist.

The cost of implementing CAR-T in clinical practice is reported only for the AOU Careggi. The IRCCS AOU BO Orsola has been intensively involved in clinical trials, and CAR-T were infused well before their use in clinical practice. The cost of implementing CAR-T in clinical practice at the IRCCS AOU BO Orsola refers only to the cost increment compared to those already incurred for the clinical studies, which amounted to €46k.

**Fig. 2:**
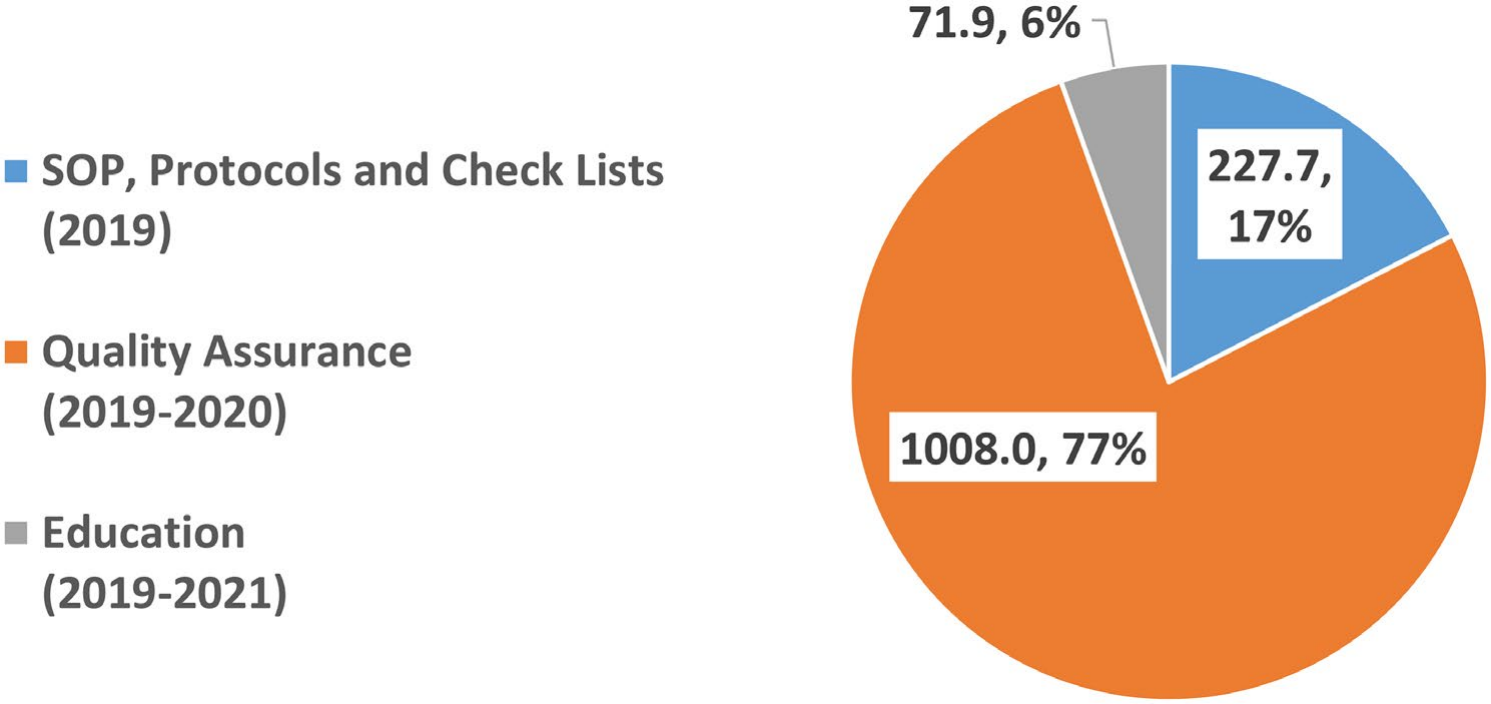
Cost of implementing the CAR-T activity in clinical practice at the AOU Careggi (2019 − 2012). Legend: Thousands of Euros and % of the total. Source: Primary data (interviews with the CAR-T coordinators) and secondary data (e.g. personnel costs) collected at the AOU Careggi

The total cost of implementing CAR-T activity at the AOU Careggi added up to €1.31 million. Quality assurance is the main cost driver (77% of costs), followed by the SOP, protocols and check list setting (17%), and education programs for the internal staff and the referral centres (6%) (Fig. 2).

Clinical pathway costs analysis refers to 59 and 27 patients who were reported as potentially eligible for treatment by the referral centre to the IRCCS AOU BO Orsola and the AOU Careggi, respectively. Of these 88%/63% were considered eligible, 41% entered the follow-up, 36%/33% survived the fourth month of follow-up (21 and 9 patients, respectively). At one year of follow-up, 19% of patients managed by the AOU Careggi (5 patients) were alive.

Table 3 illustrates the mean cost of the CAR-T pathway for patients who have completed the process (follow-up of 4 and 12 months for the IRCCS AOU BO Orsola and the AOU Careggi, respectively) and all enrolled patients. The mean total cost equals €96K (IRCCS AOU BO Orsola)-€106K (AOU Careggi) and €48K (IRCCS AOU BO Orsola) - €57K (AOU Careggi) for patients alive at follow-up and all enrolled patients, respectively.

**Table 3:** Mean and total cost of the CAR-T clinical pathway

Mean cost of CAR-T clinical pathway		Activities which are not remunerated through a fee-for-service/episode		Activities which are remunerated through a fee-for-service/episode	Total mean cost	Total costs
		General activities*	Activities provided to individual patients			
**IRCCS AOU BO Orsola**	Patients alive at follow-up (four months) (21 patients)	€ 1,334	€ 1,884	€ 92,815	€ 96,033	€ 2,837,511
		1.4%	2.0%	96.6%	100.0%	
	Patients considered eligible by the referral centre (59 patients)	€ 1,334	€ 1,884	€ 44,876	€ 48,093	
		2.8%	3.9%	93.3%	100.0%	
**AOU Careggi**	Patients alive at follow-up (one year) (5 patients)	€ 20,181	€ 3,928	€ 81,952	€ 106,061	€ 1,542,023
		19.0%	3.7%	77.3%	100.0%	
	Patients considered eligible by the referral centre (27 patients)	€ 20,181	€ 3,928	€ 33,003	€ 57,112	
		35.3%	6.9%	57.8%	100.0%	

*Coordination / SOP, Protocols and Check list updates / Quality Assurance, Audits

Source: Primary data (interviews with the CAR-T coordinators) and secondary data (e.g. personnel costs, fee-for-episode and service) collected at the IRCCS AOU BO Orsola and AOU Careggi

At the IRCCS AOU BO Orsola, costs are mainly incurred for activities that are remunerated through a fee-for-service and a fee-for-episode (93% of total mean cost per patient enrolled). The AOU Careggi shows a much higher incidence of other costs (42% and 23% of the mean cost of all patients and patients alive at follow-up, respectively). Among other costs, the main cost drivers are general activities, not referring to each individual patient. Total costs for managing 59 and 27 patients at the IRCCS AOU BO Orsola and AOU Careggi equal €2.84 and €1.54 million respectively. The AOU Careggi spent for the implementation of CAR-T and the clinical pathway of patients €2.85 million (46% and 54% for the implementation and the clinical pathway, respectively).

Table 4a and 4b illustrate the mean and total costs of each single clinical pathway phase. The activities included are the ones provided for each individual patient. Infusion-related hospitalization accounts for 71% of the total cost at the IRCCS AOU BO Orsola (74% at the AOU Careggi) whereas 14% (13%) of costs are generated by pre-admission services and 15% (13%) by follow up.

**Table 4a:** Mean and total cost of CAR-T clinical pathway by phase: IRCCS AOU BO Orsola

Clinical pathway phases	# of patients	Mean cost per patient	%	Total cost	%
Analysis of patient eligibility	59	€54	9%	€3,164	14%
Assessment of patient eligibility	52	€858		€44,623	
Consent form management, Drug choice, Communication to the relevant pharmaceutical companies	52	€284		€14,778	
Apheresis planning	52	€2,499		€129,972	
Apheresis / Therapy Request Procedure	44	€650		€28,621	
Pre-hospitalization / Lymphocyte engineering process	36	€4,516		€162,570	
Inpatient episode: Lymphocyte reception / Hospitalization / Bridge therapy / Infusion / Monitoring / Toxicity management	29	€67,033	71%	€1,943,960	71%
Discharge	27	€226	20%	€6,101	15%
Follow-up (+ 30 days)	24	€11,623		€278,952	
Follow up (+ 120 days)	21	€6,955		€146,053	
**Total (living patients after 120 days of follow-up)**	**21**	**€94,699**	**100%**	**€2,758,793**	**100%**
**Total (patients reported as eligible)**	**59**	**€46,759**			

Source: Primary data (interviews with the CAR-T coordinators) and secondary data (e.g. personnel costs, fee-for-episode and service) collected at the IRCCS AOU BO Orsola

**Table 4b:** Mean and total cost of CAR-T clinical pathway by phase: AOU Careggi

Clinical pathway phases	# of patients	Mean cost per patient	%	Total cost	%
Patients eligibility check	27	€ 1,429	9%	€ 38,579	13%
Evaluation and confirmation of eligibility / patients enrollment	17	€ 2,771		€ 47,099	
Lymphocytoapheresis planning and execution / Lymphocyte engineering procedure and bag delivery	17	€ 1,103		€ 18,744	
Bridge therapy (radiotherapy (10) or chemotherapy (2)) and bag reception	12	€ 1,701		€ 20,417	
Outpatient prelymphodepletion and lymphodepletion	2	€ 423		€ 5,078	
Inpatient episode: Lymphodepletion and infusion	12	€ 61,812	74%	€ 741,740	74%
Follow up (+ 30 days)	11	€ 3,373	17%	€ 37,103	13%
Follow up (+ 60 days)	11	€ 1,132		€ 12,449	
Follow up (+ 90 days)	11	€ 2,388		€ 26,265	
Follow up (+ 120 days)	9	€ 547		€ 4,921	
Follow up (+ 180 days)	9	€ 2,678		€ 24,100	
Follow up (+ 365 days)	5	€ 4,129		€ 20,644	
**Total (patients living after a follow-up of one year)**	**5**	**€ 83,484**	**100%**	**€ 997,138**	**100%**
**Total (patients reported as eligible)**	**27**	**€ 36,931**			

Source: Primary data (interviews with the CAR-T coordinators) and secondary data (e.g. personnel costs, fee-for-episode and service) collected at the AOU Careggi

**Fig. 3:**
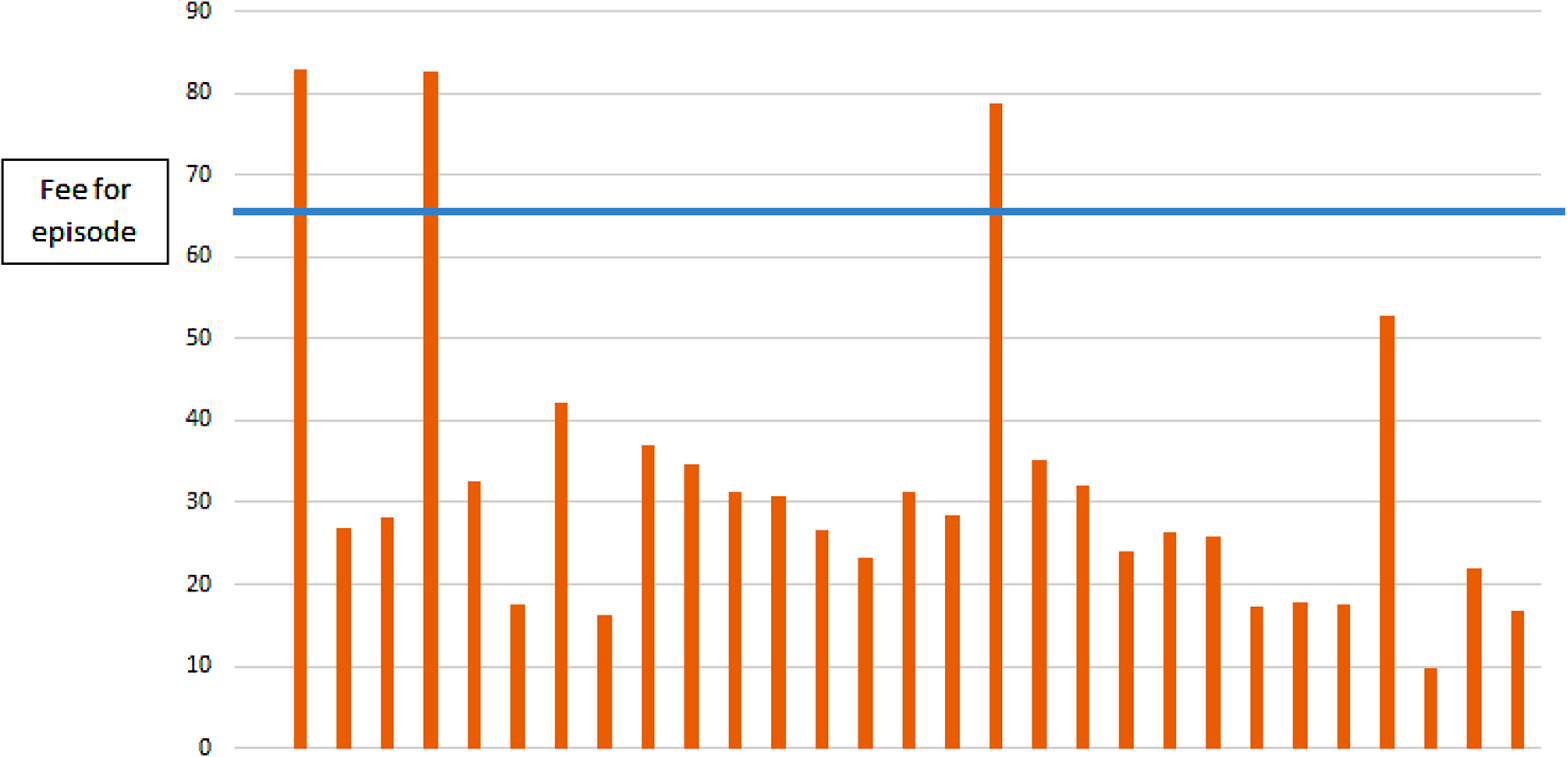
Cost per episode of hospitalization compared to fee-for-episode at the IRCCS AOU BO Orsola. Legend: Euros, thousands. Source: Patient-level secondary data (length of stay; cost per hospitalization day; diagnostic procedures) collected at the IRCCS AOU BO Orsola

Finally, we compared the hospitalisation cost sustained by the IRCCS AOU BO Orsola for each patient to the relevant fee-for-episode. In general, the cost is lower than the corresponding fee (€67K). We also observed a huge variability of unit costs per patient, determined by variations in the length of hospitalization, the use of intensive care units and laboratory diagnostic services (Fig. 3).

## Discussion

To the best of our knowledge, this is the first study investigating the organizational impact and cost of implementing a CAR-T team and the full cost of managing the clinical pathway for CAR-T patients. The only other available evidence reported, for one Portugese centre [31] and some Italian hospitals [32], the cost of services provided to each individual patient, but not the implementation costs and other costs sustained for running the CAR-T team.

The implementation costs of CAR-T equal €1.31 million at the AUO Careggi. Quality assurance activity accounts for 77% of these costs.

The mean cost per patient entering the CAR-T pathway and surviving at follow-up ranges from €48K to €57K and from €96K to €106K respectively. The latter is 30% of the list price of axicabtagene ciloleucel and tisagenlecleucel (the actual price is not public, since the discount is confidential and the impact of the outcome-based agreement is unknown). The median cost per patient treated with CAR-T (excluding the CAR-T cost) in the Portugese study is much lower (€10.6K), but the costs are not comparable, since our study estimated the full cost (including general activities not provided to one specific patient) and overhead costs.

Differences between the costs of managing CAR-T patients between the two centres depend on:

- a different year of CAR-T implementation: the higher proportion of costs for general activities at the AOU Careggi depends primarily on the different organisation of these activities. Another reason could be the more recent implementation of CAR-T, that provides for more frequent adjustment of coordination, SOP, protocols and check lists;
- the mix of healthcare professionals involved, their seniority and, consequently, associated mean costs;
- management of the clinical pathway: for example, follow-up is differently allocated between the healthcare centre that manages the gene therapy infusion and the referral centre;
- the healthcare setting in which the process is managed, with particular reference to the pre-admission and follow-up phases;
- the dimension of overhead costs allocated with the CAR-T activity.

Despite these differences, the largest proportion of costs of the clinical pathway (more than 70%) in both centres is generated by the fees for hospitalization and infusion of gene therapy. The insight into the cost of hospitalization at the IRCCS AOU BO Orsola revealed that the actual cost greatly varies across patients, and that it is in general lower than the fee-for-service.

The study has three main limitations. First, it is exploratory in nature and the two selected hospitals should be considered “case-studies” of a hospital which hosted a clinical trial on CAR-T and a hospital that implemented a CAR-T activity, once the first CAR-T was launched into the market: any extrapolation from this two case-studies to the national level should be considered arbitrary. As it was mentioned before, we have tried to involve one private large hospital, but it was unable to collect data. Secondly, we estimated the time spent by the staff on activities which are not remunerated through fees on the grounds of interviews with the CAR-T patient management coordinators. As already mentioned, the use of a more robust and analytical approach, such as that of time-motion, was not feasible. Finally, despite the fact that cost estimation relied on the same approach, primary and secondary data are not fully comparable. For example, overhead costs incidence was notably different and the follow-up period was different.

## Conclusions

Despite these limitations, this research highlighted the relevant organisational and economic impact of CAR-T implementation and clinical pathway, with important policy implications.

Implementing a CAR-T program requires a huge investment, and the launch of new CAR-T and/or the extension of their indications, may require additional investments, since economies of scale and scope cannot be indefinitely exploited. Important resources should have been or should be allocated to healthcare organisations to make this investment sustainable.

Managing CAR-T patients is also costly. On the one hand, some activities are not remunerated through fees for service / episode. On the other side hospitalization costs are for most patients lower than fees provided by the regional government to hospitals. We are not aware of the net impact on costs of these two aspects, but the clinical pathway is decidedly complex and bundled payments (or global budget) could possibly be a better option compared to fee-for-episodes and fee-for-services. This option could also assuage the inconsistencies inherent in using the same fee-for-episode for hospitalizations where costs can be very different. It’s worth mentioning that in Italy hospitalization episodes are classified through the Diagnostic-Related-Group system, that was designed to minimise intra-DRG cost variability.

Our analysis provides useful information for the implementation of the CAR-T activity costs within an in-house (or decentralised) CAR-T program. We are aware that some of this costs would be not incurred with a decentralised approach (e.g. contracting costs), but the latter would mostly affect the cost of therapy.

The relevance of the research goes beyond the limited evidence on this topic and the policy implications. The two healthcare organisations involved were provided with an operational tool that will allow them to update the organizational and cost impact data as new patients are included in the CAR-T pathway. This represents a fundamental step to evaluate the future economic sustainability of therapies with high organizational impact.

## Data Availability

The datasets generated and/or analysed during the current study are not publicly available since the IRCCS AOU BO Orsola and the AOU Careggi are the owners, but are available from the corresponding author on reasonable request.

## Abbreviations

ABC: Activity Based Costing
ALL: Acute Lymphoblastic Leukaemia
AOU: Azienda Ospedaliero-Universitaria (Public Teaching Hospital)
AOU Careggi: Azienda Ospedaliero-Universitaria Careggi di Firenze
CAR-T: Chimeric antigen receptor T cells
Cergas: Centre for Research on Health and Social Care Management
CRS: Cytokine Release Syndrome
GMP: Good Manufacturing Practice
HTA: Health Technology Assessment
IRCCS: Istituto di Ricovero e Cura a Carattere Scientifico (Public Clinical Research Hospital)
IRCCS AOU BO Orsola: Azienda Ospedaliero-Universitaria di Bologna
JACIE: Joint Accreditation Committee - ISCT & EBMT
NUB: Neue Untersuchungs und Behandlungsmethoden (New examination and treatment method)
PMSI: French Medical Information Systems Program
SOP: Standard Operating Procedures
